# Are we doing enough to control infection risk in Australian small animal veterinary practice? Findings from a mixed methods study

**DOI:** 10.3389/fpubh.2024.1388107

**Published:** 2024-11-12

**Authors:** Angela Willemsen, Rowland Cobbold, Justine Gibson, Kathryn Wilks, Simon Reid

**Affiliations:** ^1^School of Public Health, The University of Queensland, Herston, Brisbane, QLD, Australia; ^2^School of Veterinary Science, The University of Queensland, Gatton, Brisbane, QLD, Australia; ^3^Infectious Diseases and Medical Microbiology, Sunshine Coast University Hospital, Birtinya, QLD, Australia

**Keywords:** small animal, risk management, infection prevention and control, standard precautions, transmission precautions, zoonoses, multidrug resistant organisms

## Abstract

**Background:**

Managing risk effectively within small animal veterinary practice is integral for staff, patient and client safety. Veterinary personnel are exposed to many risks, including bites, scratches, sharps injuries and exposure to zoonotic diseases and multi-resistant organisms. Patients may also be exposed to healthcare-associated infections, including multi-resistant organisms. While veterinary owners/managers have a duty of care under legislated Workplace Health and Safety requirements, all staff have a responsibility to contribute to assessing and minimizing risk. The application of standard and transmission precautions will help with risk minimization. This study aimed to determine how small animal veterinary staff understand and perceive infection prevention and control risk and to provide recommendations to assist with risk mitigation.

**Methods:**

A mixed methods design was used. A digital questionnaire was administered to small animal veterinary staff in Australia to identify knowledge, attitudes and practices of risk related behaviors. Follow up focus groups were conducted with small animal practitioners to explore factors supporting and preventing veterinary staff from implementing recommended practices identified in the questionnaire.

**Results:**

Small animal veterinary staff acknowledged they participated in many high-risk activities, including recapping needles and eating and drinking in patient care areas. Injuries were common, with 77% of staff receiving a bite or scratch, and 22% receiving a sharps injury in the preceding six months. Less than one in five of these incidents was reported. Staff agreed effective infection prevention and control was the responsibility of all staff, but a designated staff member should take responsibility for managing it. The practice owner/manager was integral to supporting and promoting recommended strategies, contributing to a positive workplace culture and improving safety for staff and patients.

**Conclusion:**

Small animal veterinary staff have some understanding of how to identify, report, manage and mitigate risk but were limited by their knowledge of infection prevention and control principles.

## 1 Background

Managing risk is an integral component of how veterinary professionals perform their role and complete activities every day. Effective risk management contributes to the wellbeing of patients, veterinary staff, animal owners and their families, referred to as clients from hereon in. The nature of veterinary work exposes staff to diverse personal infectious risks, including bites and scratches, sharps injuries, zoonotic diseases, multi-resistant organisms (MRO), and interactions with clients who may have infectious diseases (1). Managing these risks may occur formally, using frameworks and staff discussions, or informally (2) and perhaps intuitively, using knowledge of disease transmission, animal management and infection prevention and control (IPC) principles (3).

In Australia, risk is defined by Safe Work Australia as; “The possibility harm (death, injury, illness) might occur when exposed to a hazard” [(4) p. 27]. The responsibility for providing a safe working environment is a requirement of the business owner and all employees, both in terms of professional and legislative responsibilities (2, 5, 6). The diversity and potential for hazards within veterinary practice necessitates a workplace with good IPC knowledge and a positive safety culture.

Risks must be identified and controlled for a workplace to be as safe as practicable for all humans and animals. For risks that include infectious hazards, staff must have a good understanding of IPC principles and their effective application. Studdert et al. (7) defines infection control as, “the utilisation of procedures and techniques to reduce the spread of infection, particularly nosocomial infections.” The Australian Commission on Safety and Quality in Health Care (ACSQHC) adopts a broader approach and recognizes that “effective IPC reduces the risk of transmission of infections between patients, healthcare workers and others in the healthcare environment” (8). This is achieved through evidence-based activities based on core IPC principles, which can be adapted to each context, including veterinary practice (6).

Infection prevention and control principles comprise standard and transmission precautions (see Table 1). Standard precautions are IPC practices implemented for “the treatment and care of all patients” (6), that is, every contact with every patient, regardless of perceived or confirmed infectious status. Transmission-based precautions are additional precautions instituted for known or suspected colonized or infected patients, where standard precautions alone may be inadequate (1, 2, 6). Transmission based precautions include:

- Contact precautions via direct (person to person, person to animal, animal to animal, animal to person) or indirect transmission (transfer of microorganisms from person or animal to an object, surface or equipment),
- Droplet precautions where droplets may be found on surfaces from coughing, sneezing or procedures generating droplets, and
- Airborne precautions where microorganisms may be suspended in the air or dispersed via air currents.

**Table 1:** Standard and transmission precautions with examples of when they may be implemented within the veterinary context (1, 2, 6).

Standard precautions with examples	
Perform hand hygiene	Applying alcohol-based hand rub (ABHR) before patting dog arriving at reception Washing hands with soap and water after picking up feces when walking dog for toilet break
Use of Personal Protective Equipment (PPE)	Performing hand hygiene before donning disposable gloves Donning disposable gloves and disposable plastic gown when examining skin lesions on a dog with pyoderma Disposing of disposable gloves and plastic gown Performing hand hygiene
Perform routine environmental cleaning	Use of a checklist to ensure all equipment and environmental surfaces are cleaned regularly according to risk category
Use and management of sharps	Immediately disposing of needle and syringe into sharps container after vaccinating a cat
Reprocessing of reusable medical devices/equipment	Cleaning, disinfecting and sterilizing (if appropriate) of reusable items, including pulse oximeter probes, instruments
Respiratory hygiene and cough etiquette	Requesting a client to use disposable tissues and use ABHR after they sneeze
Aseptic technique	Perform hand hygiene and use PPE (for example, disposable gloves and plastic gown if known MRO) as needed Establish an aseptic field Maintain aseptic field while attending to wound dressing (cover wound if known MRO) Ensure animal cannot remove wound dressing Dispose of waste, environmental cleaning and perform hand hygiene
Waste management	Handle and store non-clinical waste safely with the use of disposable gloves and ABHR pre and post glove use
Handling of linen	Discard heavily contaminated bedding (wrap in bag, secure and dispose according to council regulations)
Transmission precautions with examples	
Contact	Dog with confirmed methicillin-resistant *Staphylococcus pseudintermedius* admission Admit into isolation ward Use of PPE (disposable gloves and plastic gown) Shared equipment – select single use, if possible, otherwise limit use. Ensure item able to be cleaned and disinfected Environmental cleaning – Neutral detergent and veterinary disinfectant Visitors – may need to be limited if immunocompromised or unable to comply with PPE
Droplet	Cat sneezing (upper respiratory tract infection) Avoid waiting in waiting room Triage to isolation room if possible PPE – Disposable plastic gown Perform environmental cleaning of surfaces when consultation completed
Aerosol	Dental procedures Use of PPE (masks, gloves, eye shields, gowns)
Vector borne	Pest management control program in place

The Australian Veterinary Association (AVA) Biosecurity Guidelines (2) and the Canadian IPC Best Practices for Small Animal Clinics (1) include vector borne transmission within their guidelines. They include vectors such as mosquitoes, ticks, and rodents that may be responsible for disease transmission via direct (biting) or indirect (mechanical) methods (1, 2).

Healthcare-associated infections (HAIs), also referred to as hospital associated or nosocomial infections, are infections acquired by patients while they are hospitalized. While the term was first applied within medical settings, it is now frequently applied to animal patients within a veterinary clinical context. Healthcare-associated infections in small animal practice may include surgical site infections, urinary tract infections, bloodstream infections, pneumonia and infectious diarrhea (9). They contribute to increased morbidity, length of hospital stay, mortality and costs of care (10). Healthcare-associated infections may also lead to animals being in pain (11), and, if zoonotic, place human health at risk (10, 12). Additionally, staff and their practices may be impacted financially and professionally, particularly if clients express dissatisfaction (9). Hand hygiene is the most important measure to reduce the risk of HAIs (1, 2, 6, 10).

There is a greater level of evidence supporting IPC in human healthcare than in veterinary healthcare settings. Despite this, the evidence supporting the need for good IPC practices to be understood, taught and embraced, is clear. The aims of this study are to determine how small animal veterinary staff understand and perceive IPC risk and to provide strategies and recommendations to mitigate the risks to provide a safer workplace.

## 2 Methods

### 2.1 Research design

An exploratory mixed methods design was used as information, methods and tools within the veterinary IPC domain are limited (13). The results from the reviews, questionnaire and focus groups were used to design a Pragmatic Trial, which assessed hand hygiene compliance (14) with Nurse Champions participating in interviews which contributed to the trial evaluation.

#### 2.1.1 Questionnaire

**Figure 1:**
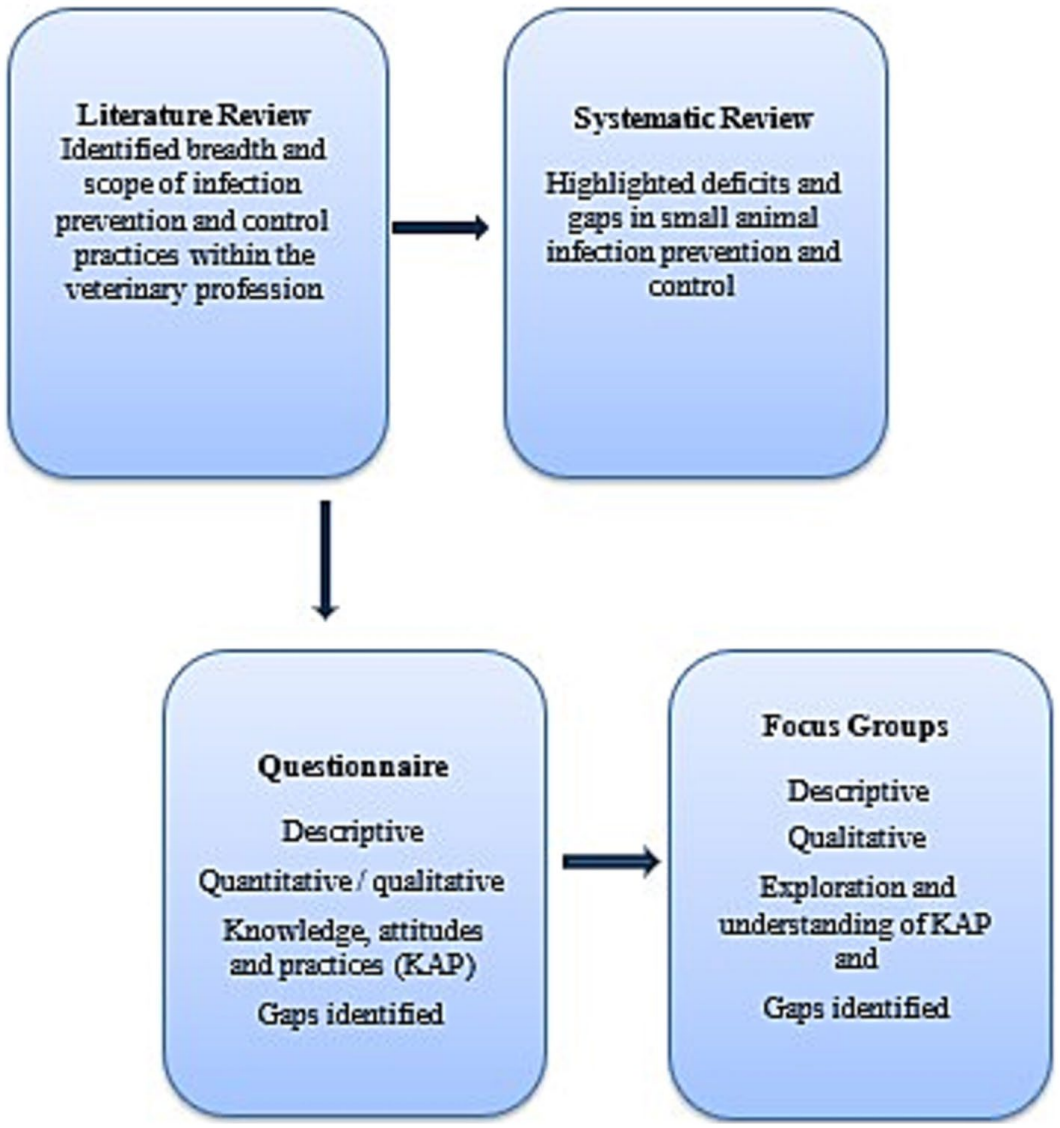
Mixed methods study design to determine the knowledge, attitudes and practices of Australian small animal veterinary staff with regards infection prevention and control.

The design of the questionnaire was based on information gained from the literature investigating IPC practices, previous veterinary surveys conducted in the field (15–24) and industry and IPC related guidelines (1, 2, 6) with the aim to understand knowledge, attitudes and practices (KAP) of IPC activities. The data gained from the KAP questionnaire prompted a need for deeper understanding of the responses, as well as further investigation into practices (Figure 1). The KAP questionnaire (Supplementary File 1) was piloted by 12 lay people and veterinary staff ineligible to participate, with minimal changes made. A total of 38 questions were included comprising a range of closed, Likert scale, short answer and open responses. Questions related to IPC practices included: hand hygiene and environmental cleaning; risk behaviors including zoonotic risk; available resources; and demographic data.

An electronic platform, UQ Checkbox®, was used to distribute the questionnaire to practicing Australian small animal veterinary staff through AVA newsletter mail outs and veterinary only social media pages (e.g., Facebook®). Individuals were encouraged to share the questionnaire link within their own networks. Respondents could not proceed until they specified consent on the digital questionnaire. The questionnaire remained open from 10 December 2017 to 30 April 2018, with regular reminders sent via all communication channels.

#### 2.1.2 Focus groups

A series of focus groups was conducted to expand on the knowledge gained from the questionnaire and to better understand how risk was perceived and managed in the workplace. The focus groups used a semi-structured interview guide (Supplementary File 2) based on questionnaire responses. The areas examined included the definition of terms used in IPC, clarification and explanation for practices performed, barriers to performing IPC and continuing education. A behavioral scientist reviewed the risk perception and focus group questions and assisted with identifying the minimum number of groups (three) required to reach saturation (25).

Staff volunteering from small animal veterinary practices were recruited through a corporate veterinary organization which volunteered to be involved and consented to their staff contributing to the trial. Corporate veterinary practices (total number undisclosed) within Queensland were emailed by the corporate executive team with those interested contacting the researcher. Focus groups enable insights into participants’ experiences, perceptions and behaviors and provide contextual information that may not be captured through other methods (26). A pilot and three focus groups were conducted, with one focus group conducted via telephone as participants were located in a remote area of Queensland (27). The pilot and the two other focus groups were conducted in urban areas within south-east Queensland. Participation was voluntary, and each participant provided written consent before participating. Focus groups lasted 60–90 min and were audio recorded, with handwritten notes taken by the researcher (AW). Questions were asked in a neutral manner to limit conformity bias, that is, participants providing responses which may be considered socially acceptable to the researcher or other participants (28). The researcher limited observer bias by not interjecting until participants had completed speaking and by not pre-empting results until all focus groups were completed (26).

The study was conducted with ethical clearance from The University of Queensland Health Research Ethics Committee (Approval number 2017001577).

### 2.2 Data analysis

Data were analyzed using mixed methods (qualitative and quantitative) analysis. This approach provides a more comprehensive understanding of the research question and provides greater credibility to the data (25). Questionnaire data were extracted and Microsoft Excel® (2016 Microsoft Corporation) was used to store, clean and perform descriptive analysis. This allowed greater understanding of the data and provided insights into data distribution and relationships. The statistical significance of any differences in responses among different groups was determined using the Chi-square test at a 95% level of confidence. All statistical analyses were performed using R software (29). Deductive thematic analysis, where data is explored with specific themes or concepts based on prior research, was used to analyze the open-ended questions. This is a qualitative research method which identifies, analyses and interprets themes (patterns) in data collected through focus groups or free text commentary. The questionnaire included free text questions allowing respondents to elaborate on their responses. Open ended responses were collated and themes ascribed (25, 26).

The recorded audio discussions from the focus groups were manually transcribed by the researcher. Familiarization of the data was achieved through several readings and systemic coding related to the research questions. As data was coded, it was placed into one or more categories. Thematic analysis was performed with the researcher working iteratively and deductively (25) until recurring concepts and key themes were identified. Qualitative researchers provided support with refining themes and insights into interpretation. All data were copied into NVivo 12 Pro (30) used for organizing and analyzing qualitative data. Qualitative data analysis software allowed sorting of descriptive codes and themes in the data and provided a visual model of themes and relationships. Microsoft Word 2016® was used to organize themes, include analytical insight and identify key quotes from questionnaire free text and focus group responses. This process provided an audit trail for the researcher to review if required.

## 3 Results

### 3.1 Questionnaire

Digital questionnaire responses, comprising 163 small animal veterinary staff (119 veterinarians and 45 veterinary nurses (VNs) and veterinary technicians^1^), were received. The denominator varied as not all respondents completed all the questions. General practice (GP) (62%, 28/45) was the most common employment location for VNs with the remaining (38%, 17/45) respondents working in specialist and/or emergency facilities. Approximately two thirds of respondents (66%, 113/163) worked full time, defined as 38 h or more/week. Demographic data is presented in Table 2.

**Table 2:** Demographic data describing small animal veterinary staff who responded to the knowledge attitudes and practices questionnaire and follow up focus group participants.

	Questionnaire respondents *N* (%)	Focus group respondents *N* (%)	Focus group breakdown			
Occupation			1	2	3	4
Veterinarian	119 (72.6%)	17	9	3	3	2
Veterinary Nurse/Technician	45 (27.4%)	9	0	3	3	3
Student nurse	0	1	0	1	0	0
Practice Manager	0	1	0	0	0	1
Practice type						
General Practice	83 (70%)	100%				
Specialist/Emergency center	27 (23%)					
Shelter	1 (1%)					
Mobile practitioner	1 (1%)					
Not reported	7 (5%)					

It was not possible to estimate the response rate for the questionnaire because the number of registered and practicing small animal veterinary staff in Australia is not available. In addition, the numbers of VNs is only available in Western Australia.

### 3.2 Focus groups

The four focus groups consisted of 28 participants (females =26, males =2). The first (pilot) focus group of veterinary staff was conducted at a regular journal club gathering coordinated by the Corporate Practice Education manager. The other three focus groups were all conducted during the scheduled lunch break. Veterinary staff continued to provide inpatient care and respond to phone calls and client enquiries throughout the discussion. One VN elected not to participate after reading the participant information and consent form.

Themes identified in the questionnaires related to knowledge, beliefs and risks of transmission of infectious agents between animals and humans, as well as preventive measures to reduce pathogen transmission and risk for staff and animals. There were five broad focus group themes identified: the complexity of IPC, barriers and resource availability to IPC, workplace culture, IPC training, and, support and ownership of IPC. The statistical results from the questionnaire provide evidence for some of the statements offered by participants. Personal and patient related risks were discussed across all five themes.

### 3.3 Personal risk

Knowledge and practices related to personal risk within the workplace were quantified in the questionnaire. Focus groups provided insight into why and when some of these risks were performed, and how they may be mitigated.

#### 3.3.1 Transmission of infectious agents

Questionnaire respondents were asked which IPC strategy was the most effective in reducing disease transmission in small animal practice in three scenarios: human-to-animal, animal-to-human, and animal-to-animal. Strategies provided included hand hygiene (with soap and water or alcohol-based hand rub), PPE, isolation rooms, separate treatment and examination rooms, antimicrobial use, disinfectant use, or none of the offered options (Table 3). Hand hygiene was identified as the most effective IPC strategy in reducing human-to-animal transmission (72%, 103/141) and animal-to-animal transmission (47%, 66/138). Reducing the risk of animal to human disease transmission placed almost equal emphasis on the use of PPE or performing hand hygiene as reducing the risk of animal-to-human disease transmission. Disinfectant use to reduce animal to animal transmission was selected by a quarter of participants. Animal-to-animal disease transmission revealed the most varied responses with isolation rooms, PPE use, and separate treatment rooms considered suitable risk minimization strategies. Antimicrobial use was not considered a strategy to reduce disease transmission by any respondents. Analysis of questionnaire responses identified an understanding of pathogen transmission with 95% (151/159) of participants acknowledging that pathogens may be transmitted from humans to animals and almost three-quarters (72%, 115/159) expressing strong agreement that they were expected to demonstrate rigorous IPC practices in the workplace.

**Table 3:** Most effective strategies selected by participants to reduce the risk of disease transmission in three scenarios.

	Hand hygiene	Personal protective equipment	Isolation rooms	Separate rooms	Antimicrobial use	Disinfectant use
Human Animal	72% (*n* = 103)	22% (*n* = 31)	NIL	1% (*n* = 1)	NIL	4% (*n* = 6)
Animal Human	47% (*n* = 66)	49% (*n* = 68)	NIL	NIL	NIL	3% (*n* = 4)
Animal Animal	47% (*n* = 66)	7% (*n* = 10)	15% (*n* = 21)	6% (*n* = 8)	NIL	25% (*n* = 36)

The most commonly selected strategy is shaded dark grey, and the next most commonly selected strategy is shaded light grey.

#### 3.3.2 Zoonotic disease

Questionnaire responses revealed that about 36% (56/158) of veterinary staff identified the risk of acquiring a zoonotic disease within small animal practice as a concern. Acquiring methicillin-resistant *Staphylococcus* spp. from their patients was considered a risk by just over a quarter (27.4%, 43/157) of respondents, with almost 28% (44/158) noting veterinary staff should be screened. More veterinarians (39%, 44/114) reported screening was unnecessary compared with VNs (18%, 7/40) (*p* = 0.0344). This difference was statistically significant at the 95% confidence level, chi square.

Veterinary staff were asked if they had contracted a zoonotic disease in the preceding 12 months. A total of 13% (15/114) of veterinarians and 15% (6/41) of VNs reported they had contracted a zoonotic disease. The most common pathogens reported were dermatophytosis (*n* = 17) along with ‘Mycoplasma’ (*n* = 2), *Pasteurella multocida* (*n* = 1), and *Bartonella henselae* (*n* = 1). Several unconfirmed and nonspecific diseases were reported, including gastroenteritis (*n* = 4) (one confirmed as *Salmonella* and an Infectious Diseases Physician suspecting another to be *Giardia*), chest infection (*n* = 1), and dermatitis (*n* = 1). About 29% (44/153) of respondents indicated awareness of a colleague who had contracted a zoonotic disease in the preceding 12 months.

#### 3.3.3 Eating and drinking in the workplace

Questionnaire respondents reported the taking of food and drink into patient care areas by veterinary staff as common, with only 9% (15/159) never taking food or drinks into patient care areas. Almost 50% (74/159) of veterinary staff (veterinarians 56/115; VNs 18/44) admitted taking food and drink regularly into patient care areas. All focus group participants regularly ate and drank in clinical areas, often due to being unable to take a regularly scheduled meal break. Water bottles and hot beverages were commonly accessed in consultation and treatment rooms and during high exposure risk procedures such as dentals.

*“…we are not fantastic at taking a regulated break, or any regulated breaks, so we tend to do a lot of things on the job, and that’s probably not fantastic as far as infection control…” (GP Veterinarian Focus Group 3A).*

*“We make sure that we cover any food that’s out whenever there’s a dental going on” (GP Veterinarian Focus Group 7A).*

*“…she’ll be completely dressed…mask, eye protection…another layer on over the top of her normal scrub top and gloves …and take a sip” (GP Veterinarian Focus Group 5A discussing a veterinarian drinking coffee while performing a dental procedure).*

#### 3.3.4 Injuries – bites, scratches, sharps

A total of 164/167 questionnaire respondents (98%) reported one or more bite, scratch or sharps injury in the past six months. Bites and scratches were the most common injury reported (77%, 128/167), with most bites and scratches affecting hands and arms, and one case including a bite to the face. Scratches were the most frequent (46%, 76/167), inflicted mainly by cats [35% (of total injuries), 59/167]. Only 22% of veterinary staff [veterinarians (26/118) and VNs (10/46)] completed an incident report in their workplace.

*“I was bitten on my hand by a cat. The bite was over the joint of my thumb, and I needed antibiotics” (GP Veterinarian, Respondent #1113).*

Questionnaire results indicated that sharps injuries had been sustained by 57% (93/164) of veterinary staff in the preceding six months. Needle stick injuries comprised 20%, 27/167 of total injuries. Recapping needles was a common practice, with only 2% (2/115) of veterinarians and 2% (1/45) of VNs never recapping a needle during a normal working day. Recapping needles ‘more than five times’ a day or ‘every time’ was reported by 74% (85/115) of veterinarians and 76% (34/45) of VNs.

*“Needle stick when collecting blood – not quite sure how it happened” (GP Veterinarian, Respondent #755).*

The uncapping of needles using teeth was a common behavior among questionnaire respondents and focus group participants. A total of 78% (93/115) of veterinarians and 69% (31/45) of VNs admitting to this practice in the questionnaire. Focus group participants reported this behavior was perceived as necessary because of the challenges of working with animals who could be unpredictable. Requesting assistance from other staff to restrain animals was not always possible or feasible when the task needed to be completed quickly.

### 3.4 Patient related risk

#### 3.4.1 Healthcare-associated infections

Results of the questionnaire showed that some veterinary staff believed clients were concerned about their pets acquiring diseases from other animals while receiving care. Only 31% (36/114) of veterinarians and 20% (8/40) VNs believed their clients were concerned. There was agreement between veterinarians and VNs, with almost 72% (113/158) overall perceiving clients were NOT concerned about their pets contracting a MRO.

#### 3.4.2 Isolation areas

Questionnaire findings identified the need for a dedicated isolation area for infectious patients with suspected or confirmed canine parvovirus (CPV), canine infectious respiratory disease complex (CIRDC) or feline upper respiratory tract disease (FURTD). Almost 80% (*n* = 117/150) of respondents reported that a dedicated area was available yet may not be adequate to limit pathogen transmission. The remaining respondents (33/150) reported use of non-purpose-built spaces where suspected or known infectious patients were placed. These locations included existing rooms within the practice (Table 4), cohabiting of animals (such as infected dogs in the cat ward and vice versa, including the use of a mobile cage), or referring animals to another veterinary practice. One focus group identified the use of the staff bathroom as an isolation area which required staff to use the clients’ bathroom.

**Table 4:** Placement of confirmed or suspected infectious animals when an isolation area/room was not available in small animal veterinary practice.

Location of the isolation area	Total
Consultation or other room “In separate consultation room or as far away as we can in the treatment area” (GP Veterinarian, #1186) “Small separate room behind radiology room with other storage in the room” (GP Veterinarian, #565) “Separate room at back of clinic (not a dedicated room) contains washing machine and freezer” (GP Veterinarian, #616) “Far end of cage bank” (GP Veterinarian, #1085)	11
Radiology room	2
Laundry “Confined to a separate mobile cage in laundry” (GP Veterinarian, #710)	1
Staff bathroom	1
Grooming area	1
Outside area of veterinary practice	1

Focus group participants reported that the location of isolation rooms was not always practicable. One practice located the isolation area upstairs, forcing staff to carry or walk patients through the treatment room to access it. Suggestions which could contribute to the safer isolation of suspected or infectious patients were proposed by participants. These included conducting consultations outside, for animals such as dogs with suspected infectious tracheobronchitis or carrying small animals directly to a consultation room to bypass the waiting area. Some staff opted for donning an additional scrub top, which was laundered at the conclusion of the consultation and with spraying the room with a non-specified veterinary disinfectant and leaving it vacant for an unspecified period to reduce risk of infection. Lastly, the development of an isolation ward policy or protocol for staff to refer to and identify potentially infectious patients at initial contact, such as when ringing for an appointment helped plan for the patient’s arrival.

More GP (31%, 28/90) than specialist/emergency centers (8%, 3/40) did not have dedicated isolation facilities. Almost 10% (14/44) of respondents did not provide a response. Practices that had isolation areas reported that they contained dedicated equipment that remained in the room for the duration of care. Some practices also housed stray animals in the isolation areas until they were collected.

### 3.5 Risk management

#### 3.5.1 Barriers to performing hand hygiene

Focus group participants reported a lack of resources to enable efficient IPC practices to be conducted. One practice discussed the lack of hand wash at sinks used for hand hygiene, while another advised a request for wall mounted hand wash dispensers was declined because of perceived costs. Staff at another practice were reluctantly forced to use the surgical scrub sink located in the treatment room for routine hand hygiene. The surgical scrub sink at another practice did not have hot water connected.

*“I am overly relaxed with hand hygiene when handling patients I deem ‘non-infectious’ and between patients.” (#1061, Specialist Veterinarian).*

#### 3.5.2 Use of personal protective equipment

In a series of questions related to risk within the workplace, almost a quarter (23%, 35/154) of veterinary staff strongly agreed/agreed that their use of PPE was perceived by their colleagues as overcautious. Only 27% (42/154) believed clients were concerned about IPC practices.

*“Gloves, mask AND eye wear must be worn when performing any type of dental procedure” (GP Veterinarian, #1079).*

The availability and use of PPE was discussed only by focus group participants. Only one practice from the focus groups reported using P2/N95 respirators during dental procedures and some avian consultations. Staff could not recall receiving P2/N95 fit testing or fit checking instructions. The cost of required PPE was often passed onto the client. This became prohibitive in lower socioeconomic regions where clients were unable to absorb the cost, placing an additional economic burden on the practice owner.

*“Get my nurses to wear gloves more frequently, especially when handling body waste. I suspect their courses never reinforced the importance of this. It is an uphill battle.” (Specialist Veterinarian, #1121).*

#### 3.5.3 Veterinary recommended vaccinations

Questionnaire findings revealed that veterinarians had higher reported rates of vaccination than VNs with all four listed vaccines (Q fever, rabies, influenza, tetanus). Vaccination for Q Fever was higher among veterinarians (75%, 84/112) with only 46% (18/39) of VNs vaccinated. Of the veterinarians who were not vaccinated, three were from international universities, 17 had graduated before and four after 2000^2^. Eight veterinarians did not identify where they graduated from. Of the 102 who had received a Q Fever vaccination, 9% (9/102) believed they were not current and 23% (23/102) were unsure if they were current or not.

More veterinarians (38%, 42/111) than VNs (11%, 5/45) had received a rabies vaccination and of those, 60% (25/42) of veterinarians and 40% (2/5) of VNs stated they were current. The remaining veterinarians (40%, 17/42,) and VNs (60%, 3/5) were unsure of currency, were not current or did not respond. Of these, a veterinarian and a VN both stated they provided care for bats. Overall, 14% (22/155) of respondents worked in practices providing care for bats. Bats were either handled by staff who had received the rabies vaccination or by wildlife carers, presumed to be vaccinated. The exception was an additional seven of 156 (4%) respondents who had not received the rabies vaccination with one veterinarian stating they provided care to bats.

Most veterinarians (97%, 115/118) and VNs (91%, 42/46) had received a tetanus vaccination, with 68% (111/164) believing they were current. Just over half of veterinarians, 58% (69/118) and under half of VNs (48%, 22/46) had received an influenza vaccination. The COVID-19 vaccination was not available at the time of the questionnaire.

### 3.6 Infection prevention and control in the workplace

#### 3.6.1 Workplace culture

Workplace culture was defined as “what you do at the clinic” (Veterinarian Clinic A) and influenced whether or not staff implemented effective IPC practices. Changing the culture was recognized as needing to be initiated and endorsed by a manager or senior staff member. Focus group participants recognized that staff behaviors, positive or negative, influenced veterinary students observing IPC practices while on practical rotations.

*“My boss is anti PPE. Does not wear gloves or mask in dentals, does a lot w(ith) bare hands. Does not stress infection control at all” (GP Veterinarian, Questionnaire respondent #829).*

#### 3.6.2 Ownership of IPC in the workplace

There was widespread consensus among focus group participants that IPC was the responsibility of all employees. Most participants also agreed on the need for IPC to be coordinated and managed by someone in the practice. Determining who should manage IPC practices was divided between the veterinary owner/manager or the VN in charge, both of whom were identified as already having additional duties. Participants commented on the need for the person driving IPC to have a personal interest in IPC. The veterinary owner was considered essential to support and endorse the implementation of IPC activities. Reduced staffing levels and attending to the core business of patient care relegated additional activities, such as IPC, to a lower priority. A factor which may contribute to resistance within the workplace was instituting an IPC plan as a directive from executive management rather than for clinical need.

*“You need a champion. You need someone in the building who really cares and is on to it and keeping everyone in line” (GP Veterinarian 3C).*

## 4 Discussion

The application of IPC principles within veterinary practice are recommended by the AVA and endorsed with the AVA Code of Practice (3) and the AVA Guidelines for Veterinary Personal Biosecurity (2). The AVA Code of Practice acknowledges there is “no current specific legislation requiring veterinarians to develop, undertake or comply with formal infection control plans and procedures.” The code includes the legislative need to comply with Workplace Health and Safety (WHS) obligations (31). As an example, the Veterinary Surgeons Act 1936 (Queensland) (32) refers to conditions about “hygiene practices…” and regulations which may be made under the Act for “methods of hygiene and standards of cleanliness therein.” The need for more stringent IPC within the veterinary profession is promoted with the release of national strategies (33, 34) to minimize and respond to the global threat of antimicrobial resistance. Suggestions as to how IPC may be improved in veterinary practice is unclear.

### 4.1 Personal risk

These research findings demonstrate that small animal veterinary staff have an awareness of infectious risks which they may be exposed to within the workplace. Their awareness for managing or preventing these risks is inconsistent among practitioners and practices. This was evident with the perceived effectiveness of IPC strategies in reducing disease transmission between humans and animals. Animal-to-animal transmission would be considered a HAI, and transmission is often through indirect means, typically involving veterinary staff (11). Effective hand hygiene was recognized as important but less so when considering transmission between animals or from animals to humans. The use of PPE is useful but may provide the user with a false sense of security if the PPE is used incorrectly or incorrect removal results in self-contamination (35). A quarter of respondents identified disinfectant use as the second most important factor in reducing animal-to-animal disease transmission. Effective disinfectant use is reliant upon prior cleaning to remove organic matter and the use of an appropriate disinfectant at the correct dilution and contact time (1, 2, 6, 36). Disinfectant selection must consider effectiveness against pathogens. For example, feline calicivirus (FCV) is a pathogen with known resistance to many disinfectants (37).

This research identified that over a third of veterinary staff acknowledged they were concerned about contracting a zoonotic disease and about one in seven veterinary staff reported contracting a zoonotic disease in the preceding 12 months. This aligns with studies which reported incidence of zoonoses among veterinarians ranging from 10 to 25% and another reporting 45% of veterinary staff contracting a zoonotic disease while working (16, 38). This is a large number of veterinary staff who have contracted a zoonotic disease. Small animal practitioners may also be exposed to various pre-existing zoonotic pathogens when caring for small animals and wildlife. Pathogens that may be encountered include; direct contact (skin, mucous membranes, bodily fluids) (e.g., *Brucella suis*, dermatophytosis such as *Microsporum* spp., *Leptospira* spp., Australian bat lyssa virus), bites, scratches or needlestick injuries (e.g., *Bartonella henselae, Pasteurella*), respiratory droplets and aerosols (e.g., *Coxiella Burnetii* (Q fever)), or fecal-oral route (e.g., *Campylobacter* spp.) (39). Multi-resistant organisms such as methicillin-resistant *Staphylococcus aureus* and methicillin-resistant *Staphylococcus pseudintermedius* are also recognized and well documented in the literature as a risk for zoonotic infection (2). These risks are increased and compounded by staff eating and drinking in patient care areas. It is probable that the implementation of more stringent IPC practices would reduce the likelihood of these occurrences.

Additionally, emerging zoonotic diseases such as Severe Fever with Thrombocytopaenia syndrome (SFTS), are posing a threat in eastern Asia. The causative organism is a newly identified bunyavirus, *Dabie bandavirus,* with a tick thought to be the primary reservoir. Transmission may be via direct or indirect contact with veterinary staff contracting the virus from a cat confirmed through whole genome sequencing (12). Transmission of SFTS from a hospitalized dog to two cats has also occurred in this case. The source of infection was not identified, but all animals used the same ventilator in which the breathing tube had not been changed or disinfected (40). This, along with other examples such as the recent confirmation of possums acting as reservoirs for Buruli ulcer (*Mycobacterium ulcerans*) in Australia (41), supports the need for veterinary staff to employ protective measures more consistently.

The number of staff sustaining injuries from bites, scratches, and sharps is considerable, with almost four out of five staff sustaining a bite or scratch and one in five sustaining a sharps injury in the previous six months (from their recall). Only one in five incidents was reported, which was relatively low. The reporting and completion of an incident report provides a record of an injury sustained in the workplace and may be needed to identify risk areas and possible risk reduction strategies. Most injuries affected arms and hands, with impacts including pain, infection and scarring, and limiting the potential to perform other tasks such as surgery. Precautions such as the use of physical restraints and chemical sedation can help to reduce the incidence (2, 42). Other strategies such as practices achieving “Fear Free” status may contribute to calmer patients in a reduced stress environment (43). Strategies to reduce sharps injuries are more reliant on changing personal habits. It is interesting to note that respondents identified poor practices associated with sharps management. For example, this quote highlighted the common practice of unsafe needle cap removal *“…not take needle caps off with my mouth…not recap needles”* (*GP Veterinarian, Respondent #818*). In this situation, a change in practice may be achieved through placement of Australian Standard puncture resistant sharps disposal containers closer to the point of use and transitioning to safety-engineered devices, such as needleless or retractable devices, are appropriate to mitigate risk (6).

### 4.2 Patient related risk

#### 4.2.1 Healthcare-associated infections

Surveillance for HAIs in Australian small animal practices is not routinely conducted, consequently, the range of pathogens involved and prevalence remains unknown. A survey of accredited American veterinary teaching hospitals in 2007 reported that 82% (31/38) had an HAI outbreak within the previous five years, 17 (45%) had multiple outbreaks, and 12 (32%) closed sections of the hospitals to limit spread (15). A French veterinary hospital identified increased surgical site infections with *Serratia marcescens* in 54 patients (32 infected, 22 colonized). The source was found to be the chlorhexidine solution containing gauze swabs used for surgical site preparation (44). Despite the lack of formal surveillance, there are numerous anecdotal reports of HAIs in small animal practice in Australia.

This research revealed that more VNs than veterinarians perceived that clients were not concerned about their pets contracting diseases from other animals or MROs while receiving care. This perception may be due to VNs believing they are confident in the measures they take to prevent the spread of disease and how they believe they are communicating and demonstrating IPC to the public. Community members were not included in this study so this perception cannot be confirmed or refuted.

#### 4.2.2 Isolation facilities

The lack of a dedicated isolation area places other animals, and potentially staff, at increased risk of contracting pathogens. Animals with suspected or confirmed diseases such as CPV, FCV or infectious tracheobronchitis may result in transmission via contact, droplet, or air-borne routes. Co-habiting or placement of patients in other rooms increases the risk of HAIs. Placing an infectious animal outside may increase the risk of transmission via vectors (2, 39). The risk of cohabitation of dogs and cats is demonstrated in an outbreak of feline infectious peritonitis in domestic and wild cats in Cyprus where researchers found the outbreak was due to a novel and highly pathogenic recombination of a Feline and Canine Coronavirus (45). There is a clear need for dedicated, suitably equipped and accessible isolation areas. Protocols need to be established, and clear instructions provided on how to operate the isolation area.

### 4.3 Control measures

The application of standard and transmission precautions, according to the assessed risk collectively reduce the risk, and the likelihood of infectious transmission. These precautions are designed to protect staff and patients (6). The main impediments to implementing these measures include the lack of adequate resourcing, building infrastructure, and understanding regarding the acquisition of products, such as ABHR dispensers. For example, suppliers may provide free dispensers when an order of ABHR is placed. For some control measures to be effective, such as P2/N95 respirators, a fit test must be performed to ensure the correct size is selected and an effective seal is achieved (6, 46). A workplace may need to stock more than one brand of respirator to meet staff requirements (2, 46). Control measures such as hand hygiene have been discussed in greater depth in another publication (14) notwithstanding, adequate availability of hand hygiene stations are required for an essential IPC practice.

Vaccinations recommended for veterinarians, veterinary science and veterinary technician students, and veterinary nurses include influenza and Q fever, and those working with bats should also have a protective rabies titer. Tetanus and COVID-19 vaccination are also recommended for all adults (47, 48). The questionnaire identified that compliance with recommended vaccinations for veterinary staff was different between veterinarians and VNs. Veterinarians and VNs who care for bats and are not up to date, or have not received rabies vaccinations, place themselves at increased risk of zoonotic diseases. While the incidence of Australian Bat Lyssa Virus is low, the potential consequence is high (49). The disparity with Q fever vaccination was pronounced with the AVA and VNs Council of Australia advocating to increase vaccination rates for veterinary staff, particularly veterinary nurses (50). This is partly due to the requirement for university students, enrolled in courses working in agriculture or with animals, to be vaccinated for Q fever. This requirement is not a condition for Registered Training Organizations who provide VN training. Of concern is the almost one in three veterinary staff who were unaware if they were current when repeated Q Fever vaccinations are contraindicated (47, 51). Non veterinary specific vaccinations such as tetanus and influenza have less discrepancy but are still higher for veterinarians. These vaccinations are an important control measure against a number of risks, and veterinary practices should implement and maintain vaccination records for their staff (2, 47).

### 4.4 Workplace culture

The need for a positive workplace culture is integral to providing a safe workplace for staff, animals and clients. The owner/manager has a responsibility under WHS to provide a safe workplace (5). This is beyond physical measures such as the provision of PPE and also includes proper training, reporting and support. Part of this role includes demonstrating acceptable behaviors and attitudes and their willingness to not accept high risk behaviors, such as uncapping needles with teeth. Ensuring staff have scheduled meal breaks (52) will contribute toward less food and drink being consumed in patient areas, with a bonus of staff being able to take a rest.

Individual staff also need to take on responsibility for contributing to and maintaining safe practices. This includes attending training, adhering to standard and transmission precautions, and supporting other staff.

### 4.5 Why is risk poorly managed?

Risk needs to be identified and understood before it can be managed (4). Historically, IPC has been a low priority for veterinary staff, as it does not overtly contribute to profitability (36). The self-efficacy, or the certainty a person has in their ability to perform a specific task, can influence IPC (53). There is evidence that the reality of the incidence of zoonotic diseases and injuries from bites, scratches and sharps is higher than perceived by small animal veterinary staff.

A three-tiered approach is needed to reduce IPC related risk in the workplace. At an individual level, employees need to understand their responsibilities, maintain their training and education, and include IPC practices such as those recommended by the AVA (2), NHMRC IPC Guidelines (6) and the National Hand Hygiene Initiative (54). Staff also have a responsibility to speak up when unsafe or high-risk practices are observed and/or continue to occur.

From a practice and corporate level, providing a safe environment where staff feel comfortable speaking up, and action is taken to improve unsafe practices is essential. Evidence based policies and procedures should be developed, communicated and maintained. An IPC champion, with the appropriate support, authority and knowledge should be appointed to enhance uptake and continued application of IPC. Organizations should strive to develop an ethos for promoting best practice and treating IPC as an embedded element of every process (1, 2).

From a national level, responsible bodies, such as the AVA, VNs Council of Australia and State/Territory Veterinary Surgeons Boards, in collaboration with the Federal government, should actively endorse and encourage the establishment of an effective IPC framework for small animal practice and the almost 29 million pets in Australia (55). The priority should be for developing guidelines/policies to enhance understanding of standard and transmission-based precautions and for vaccinating all staff engaged in animal care.

### 4.6 Strengths, limitations, and future research

This manuscript examines factors contributing to small animal veterinary staff performing high risk behaviors. Understanding motivating factors for behavior may influence strategies to help reduce the risk. The frankness and honesty of all respondents has provided important and worthy data, as well as contributing to the growing pool of evidence. As veterinary practices volunteered for the focus groups, there is a possibility of a selection bias, meaning the veterinary staff have a greater interest in IPC. The small numbers of emergency /specialist veterinary staff and VN responses may not accurately represent these populations. While measures were taken to reduce bias for both researchers and participants, it is important to acknowledge that biases may exist. Interpretation of responses or personal biases of participants may affect how they respond to questions or interact within the group setting. Remaining vigilant to sources of bias contribute to maintaining research integrity and reliability of the research findings.

Accessing workforce data representing the number of actively employed veterinary staff was not easily obtained. The questionnaire and focus groups were conducted pre-COVID, so some of the findings may differ if this study was replicated post-pandemic, due to substantive changes in infectious risk knowledge and practices based on the COVID-19 experience (56).

A greater understanding about educating future veterinary professionals about the importance of IPC is needed to build on the findings presented in this publication. Identifying which IPC principles are taught and how students are assessed as being competent by Australian universities and Registered Training Organizations will help with determining student need, identifying gaps in the curriculum and working toward improving IPC for veterinary professionals.

## 5 Conclusion

Infection prevention and control is not regarded as a priority in veterinary practice. The lack of understanding of the principles and practices of IPC limits an individual practitioner’s ability to apply them in a veterinary setting. Progress toward implementing them within the veterinary profession will only occur when they are embedded as a core component in the veterinary curricula, including continuing professional development.

## Data Availability

The original contributions presented in the study are included in the article/Supplementary materials, further inquiries can be directed to Angela Willemsen: a.willemsen@uq.edu.au.

## Footnotes

^1^From this point, the use of ‘Veterinary nurse’ includes ‘Veterinary Technician’.

^2^Compulsory Q Fever vaccination for Australian veterinary science/technician students was introduced during the early 1990s.
