# Supplementary material for: Are we doing enough to control infection risk in Australian small animal veterinary practice? Findings from a mixed methods study
Source: Front Public Health. 2024 Nov 12;12:1388107. doi: 10.3389/fpubh.2024.1388107 (PMC11588738; doi:10.3389/fpubh.2024.1388107)
Supplement: Supplementary file 1 [file Table_1.docx]

**Supplementary File 1**

**Questionnaire for Australian small animal veterinary staff**

**Q1** Part A Thinking back on your work activity in the past 6 months, have you:

- Been bitten or scratched at work? (Yes/No/Unsure)
- Had any sharps (needle sticks or scalpel blade injuries? (Yes/No/Unsure)

Part B If YES to either question, did you complete an incident report? (Yes/No)

Part C My last injury was: _____________

Part D What were your actions for your LAST injury? (Tick all that apply)

- Nothing needed
- Washed the wound at work
- Applied light dressing at work
- Sought medical attention
- Other _____________

**Q2** In the previous 12 months have you:

- Provided unprotected mouth to mouth/nose to resuscitate any neonates?
- Advised owners to protect themselves from the risk of zoonotic disease?

(Never/Very occasionally (1-2 times/Occasionally (3-5 times)/Frequently (more than 5)/Every time)

**Q3** Reflecting on your NORMAL daily routine, during a work day, have you:

- Recapped a needle?
- Uncapped a needle with your teeth?
- Manually removed a scalpel blade?
- Worn rings to work?
- Worn hand jewellery such as watches or bangles to work?
- Taken food or drinks where animals are located?
- Cleaned the top of multi-use vials with methylated spirits before use?

(Never/Very occasionally (1-2 times/Occasionally (3-5 times)/Frequently (more than 5)/Every time)

**Q4** Are there sharps containers in the following areas? (Yes/No/Not applicable)

- Each consultation room
- Wet preparation/dental area
- Surgical room/area
- Treatment room
- Other

**Q5** Do you wear gloves when performing or assisting with the following?

- Routine consultations such as vaccination
- Oral or dental examination
- Patient with skin infection
- Vomiting and/or diarrhoea patient
- Coughing patient
- Open wounds
- Obstetric patient examinations
- Handling body fluids or waste
- Handling soiled linen

(Never/Very occasionally (1-2 times/Occasionally (3-5 times)/Most times/Every time/Not applicable)

**Q6** Part A Is there an infection control plan at your workplace? (Yes/No/Unsure)

Part B If Yes, which year was it updated? ________

**Q7** Part A Does your practice have any guidelines or standard operating procedures (SOP) for medical or other procedures? (Yes/No/Unsure)

Part B If Yes, Please list two examples of guidelines or standard operating procedures in your workplace?

Part C Do you find guidelines useful within your workplace?

(Never use them/not useful/somewhat useful/useful/very useful)

**Q8** Have you referred to any of the following veterinary resources? For any references not mentioned, please add reference name to the list. (Yes/No/Unsure/Never heard of it)

- Australian Veterinary Association (AVA) Guidelines for Veterinary Personal Biosecurity
- Australian Veterinary Association (AVA) Model Infection control plan for veterinary practices
- Appendix 15 - Australian Veterinary Association (AVA) Code of Practice for prescription and use of products which contain antimicrobial agents.
- Australian Infectious Diseases Advisory Panel (AIDAP) Practical Infection Control Guidelines (Zoetis)
- Australian Infectious Diseases Advisory Panel (AIDAP) Antibiotic prescribing detailed Guidelines (Zoetis)
- Infection Prevention and Control Best Practice for Small Animal Veterinary Clinics (Canadian Committee on Antibiotic Resistance, August 2008)
- Target – The Antimicrobial Reference guide to effective treatment (Bayer)

**Q9** Where have you learnt about infection control? (Select all relevant)

- Nowhere
- Part of my formal education
- Continuing professional education
- Self taught (internet resources, journal articles etc.)
- AVA Guidelines
- Other – Please list:

**Q10** Are you involved in any auditing for adherence to ant infection control guidelines at your workplace? (Yes/No/Unsure)

**Q11** Part A In your practice, do you know which products are used to ROUTINELY clean the floor? (Yes/No)

Part B Please provide, for each product used:

- Product name/Concentration/Action (e.g. clean/disinfect)

**Q12** In your practice, how often are floors ROUTINELY cleaned?

- Once a day
- Twice a day
- More than twice a day
- I don’t know
- Other : (free text)

**Q 13** Part A In your practice, do you know which products are used to clean/disinfect work surfaces, such as work benches and consultation tables? (Yes/No)

Part B Please provide, for each product used:

- Product name/Concentration/Action (e.g. clean/disinfect)

**Q14** Part A In your practice, do you know which products are used to ROUTINELY disinfect contaminated surfaces (e.g. Canine parvo virus, Canine Kennel Cough)? (Yes/No)

Part B Please provide, for each product used:

- Product name/Concentration/Action (e.g. clean/disinfect)

**Q15** In your practice, how often are the following surfaces ROUTINELY cleaned/disinfected?

- Consultation tables
- Radiography table
- Surgery table
- Wet prep/treatment room benches
- Waiting room seating
- Waiting room animal scales
- Small animal/cat scales
- Laboratory area/bench
- Dog and cat food bowls

(Never/Once a day/Twice a day/After every patient/Don’t know/Other (free text))

**Q16** Part A Does your practice have a DEDICATED isolation area for infectious patients (e.g. Canine parvo virus, Canine Kennel Cough, Cat Flu)? (Yes/No)

Part B If No, where do you keep your suspected or confirmed infectious patients?

**Q17** In your practice, how often is anaesthetic tubing ROUTINELY cleaned/disinfected?

- Daily
- Weekly
- After every patient
- Unknown
- Other (free text)

**Q18** Part A In your practice, do you know which products are used to routinely clean/disinfect anaesthetic tubing? (Yes/No)

Part B Please provide, for each product used:

- Product name/Concentration/Action (e.g. clean/disinfect)

**Q19** How do you know that cleaning has been sufficient? (Tick all that apply)

- Looks clean
- No visible hair
- Smells clean
- I can see the product on the surface
- Have followed clinic guidelines
- Other: (free text)

**Q20** In the most recent working week, how often did you wash your hands OR use alcohol-based hand rub (ABHR)?

- Before starting work
- Before entering consultation or treatment room
- Before touching an animal’s surroundings e.g. opening cage
- Before a procedure
- After a procedure or body fluid exposure risk
- After touching animals/patients
- After touching an animal’s surroundings e.g. closing cage
- Between patients
- Before putting gloves on
- After taking gloves off
- After attending the toilet
- After handling laundry/bedding containing animal body fluids
- Before going home at the end of the day

(Always/Mostly/Occasionally/Very occasionally/Never)

**Q21** Do you think hands should be washed or alcohol-based hand rub used:

- Before starting work
- Before entering consultation or treatment room
- Before touching an animal’s surroundings e.g. opening cage
- Before a procedure
- After a procedure or body fluid exposure risk
- After touching animals/patients
- After touching an animal’s surroundings e.g. closing cage
- Between patients
- Before putting gloves on
- After taking gloves off
- After attending the toilet
- After handling laundry/bedding containing animal body fluids
- Before going home at the end of the day

**Q22** Think back to the last time that you examined or assisted with an animal and did NOT wash your hands or use alcohol-based hand rub (ABHR). What were the main reasons?

- No ready access to basin and/or ABHR
- Too busy
- Wore gloves
- Forgot
- Not important and I have never caught anything from animals
- Other (free text)
- None of the above

**Q 23** What do you think is the single most important strategy for reducing transmission of disease in small animal practice from:

Part A Animal to Animal Part B Human to Animal Part C Animal to Human

- Hand washing/ABHR
- PPE
- Isolation rooms
- Separate treatment and examination rooms
- Antimicrobial (antibiotic) use
- Disinfectant use
- None of these

Q 24 Please indicate your level of agreement with the following statements

- I am expected to demonstrate stringent infection control practices at work
- If I use PPE, other sin my workplace think I am being over cautious
- I think that mt patients are at risk from contracting nosocomial infections from other animals
- I am concerned about acquiring a zoonotic disease from the animals in my care
- I am at risk of catching methicillin-resistant *Staphylococcus* spp. from my patients
- I think that our staff should be regularly screened for methicillin-resistant *Staphylococcus* spp.
- I believe our clients are concerned about our infection control practices
- I think clients attending my workplace are concerned about their pets getting diseases from other animals
- I think our clients are concerned about their pets contracting multi-resistant infections

(Strongly disagree/Disagree/Neither agree nor disagree/Agree/Strongly agree)

**Q25** What is your opinion on the following statement?

My risk of acquiring an infectious (zoonotic) disease from an animal is ……?

(Very unlikely/Likely/Neither likely or unlikely/Likely/Very likely)

**Q26** Do you think that infectious agents can be transmitted from humans to animals? (Yes/No)

**Q27** Part A In the previous 12 months, have you: Acquired a zoonotic disease from an animal in your care?

(Never/very occasionally (1-2 times)/Occasionally (3-5 times)/Frequently (>5 times)/Often (>10times)

Part B Please list any zoonotic diseases that you have acquired in the past 12 months and indicate whether they were confirmed or suspected (Confirmed/Suspected) + Zoonoses name

**Q28** In the past 12 months, do you know of a veterinary member that has contracted a zoonotic disease from an animal in the past 12 months? (Yes/No)

**Q29** Have you had the following vaccinations? (Yes/No) and are you current? (Yes/no/unsure)

- Q Fever
- Rabies
- Tetanus
- Influenza
- Other

**Q30** What is your occupation?

(Veterinarian – Student/General Practitioner/Specialist/Other)

(Veterinary nurse/veterinary technician/students)

When and where did you graduate? (Year/institution/Do you have a specific role e.g. Practice Manager, Infection Control) (Students to include expected year of graduation)

**Q31** On average, how many hours do you work each week?

**Q32** What is your employment status? (Full time/part time/casual)

**Q33** What is your workplace postcode? (Mobile veterinary staff include postcode of head office)

**Q34** What kind of practice do you work in ?

(General Practice/Emergency Centre/Specialist Centre/Other)

**Q35** How many veterinarians are employed in the practice where you work? (Full time is equal to 38 hours/week – E.g. 2 x full time and 1 x 20 hours = 2.5 veterinarians (Free text)

**Q36** Does your practice provide care for bats? (Yes/No)

If yes, how many staff are vaccinated for rabies?

**Q37** What is the ONE thing you do well with respect to infection control?

**Q38** What do you think you could do better with respect to infection control?
