# Supplementary material for: Are we doing enough to control infection risk in Australian small animal veterinary practice? Findings from a mixed methods study
Source: Front Public Health. 2024 Nov 12;12:1388107. doi: 10.3389/fpubh.2024.1388107 (PMC11588738; doi:10.3389/fpubh.2024.1388107)
Supplement: Supplementary file 2 [file Table_2.docx]

**Supplementary File 2**

**Semi-structured Interview Guide for Small Animal Veterinary Practices Focus Groups**

1. What do you think infection control is?

What does it mean to you? Other terms such as biosecurity? Infection prevention?

1. What are the most common IC practices that you do on a daily basis?

- Glove use
- PPE
- Needle use – how many have had sharps injuries? Why did they happen? What did you do? Incident reports?
- Who removes needle caps with teeth? Why? Have you injured yourself?
- Rings or hand jewellery
- Cleaning mobile phones, stethoscopes
- Mouth to mouth of neonates
- Cleaning vs disinfection
- Water and/or coffee in the work area
- What about wearing of scrubs outside if work? Do you consider that a risk?
- Do you think that part of your job is protecting yourself/animals/clients? If so, how?

1. What are your thoughts on hand washing?
   - Is that enough?
   - ABHR use. Why or why not? Availability?
   - When are gloves worn?
2. The practices that we have spoken about, can you give me examples of different experiences?
3. In terms of IC, can you give me some examples of how you are doing enough?

- What needs to change?
- What are some of the barriers to change in practices that you have worked during your career?
- Who should be responsible for improving IC in a veterinary practice? e.g. individual, AVA, Vet board, other
- Have you thought about this before?
- How much CPD do you participate in?
- In an ideal world, what would you do?
- What would be your priorities?
- Realistically, how much time do you have available? Every week/month/quarter?
- What information or training would make remaining current with IC easier? (videos, posters, interactive on line)

1. Given what we have talked about, is there anything else that you would like to raise?
